# 5-Benzyl-2-phenyl-6,8-dihydro-5*H*-1,2,4-triazolo[3,4-*c*][1,4]oxazin-2-ium hexa­fluoridophosphate

**DOI:** 10.1107/S1600536809018005

**Published:** 2009-05-20

**Authors:** Yumin Huang, Siping Wei, Zhen Wang, Zhihua Mao, Xiaoyu Su

**Affiliations:** a Key Laboratory of Green Chemistry and Technology of the Ministry of Education, College of Chemistry, Sichuan University, Chengdu 610064, People’s Republic of China; b The Centre of Testing and Analysis, Sichuan University, Chengdu 610064, People’s Republic of China

## Abstract

The title compound, C_18_H_18_N_3_O^+^·PF_6_^−^, is a chiral bicyclic 1,2,4-triazolium salt which contains four rings, *viz*. a triazolium, a morpholine and two phenyl rings. Analysis of bond lengths shows that the N—CH—N group in the triazolium ring conforms to a typical three-center/four-electron bond (also known as the Pimentel–Rundle three-center model). The structure is completed by a disordered PF_6_^−^ counter-ion [occupancies of F atoms 0.678 (8):0.322 (8)], which inter­acts with the main mol­ecule through weak inter­molecular P—F⋯π inter­actions.

## Related literature

For details of different C—C bond-formation reactions, see: Fisher *et al.* (2006 ▶); Kerr *et al.* (2002 ▶); Knight & Leeper (1998 ▶); Readde Alaniz & Rovis (2005 ▶); Ma *et al.* (2008 ▶).
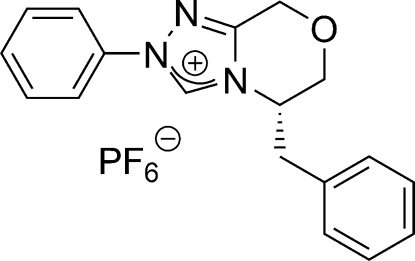


## Experimental

### 

#### Crystal data

C_18_H_18_N_3_O^+^·PF_6_^−^
*M* *_r_* = 437.32
Monoclinic, 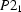

*a* = 11.4054 (13) Å
*b* = 8.1243 (9) Å
*c* = 11.8593 (14) Å
β = 118.678 (2)°
*V* = 964.09 (19) Å^3^
*Z* = 2
Mo *K*α radiation
μ = 0.21 mm^−1^
*T* = 297 K
0.53 × 0.42 × 0.32 mm

#### Data collection

Bruker SMART CCD area-detector diffractometer
Absorption correction: multi-scan (*SADABS*; Sheldrick, 1996 ▶) *T*_min_ = 0.89, *T*_max_ = 0.93
5505 measured reflections
3406 independent reflections
2984 reflections with *I* > 2σ(*I*)
*R*_int_ = 0.022

#### Refinement

*R*[*F* ^2^ > 2σ(*F* ^2^)] = 0.039
*wR*(*F* ^2^) = 0.114
*S* = 1.11
3406 reflections
318 parameters
31 restraints
H-atom parameters constrained
Δρ_max_ = 0.20 e Å^−3^
Δρ_min_ = −0.20 e Å^−3^
Absolute structure: Flack (1983 ▶), 1368 Friedel pairs
Flack parameter: 0.01 (10)

### 

Data collection: *SMART* (Siemens, 1996 ▶); cell refinement: *SAINT* (Siemens, 1996 ▶); data reduction: *SAINT*; program(s) used to solve structure: *SHELXS97* (Sheldrick, 2008 ▶); program(s) used to refine structure: *SHELXL97* (Sheldrick, 2008 ▶); molecular graphics: *SHELXTL* (Sheldrick, 2008 ▶); software used to prepare material for publication: *SHELXTL*.

## Supplementary Material

Crystal structure: contains datablocks global, I. DOI: 10.1107/S1600536809018005/bg2257sup1.cif

Structure factors: contains datablocks I. DOI: 10.1107/S1600536809018005/bg2257Isup2.hkl

Additional supplementary materials:  crystallographic information; 3D view; checkCIF report

## Figures and Tables

**Table 1:** Hydrogen-bond geometry (Å, °)

*D*—H⋯*A*	*D*—H	H⋯*A*	*D*⋯*A*	*D*—H⋯*A*
P1—F1⋯*Cg*1^i^	1.57 (1)	3.03	4.235 (11)	132
P1—F2⋯*Cg*1	1.57 (1)	3.19	4.102 (11)	115
P1—F2′⋯*Cg*1	1.50 (1)	2.93	4.102 (11)	133

Symmetry code: (i) 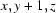. *Cg*1 is the centroid of the N1/N2/C8/N3/C7 ring.

**Fig. 1.**
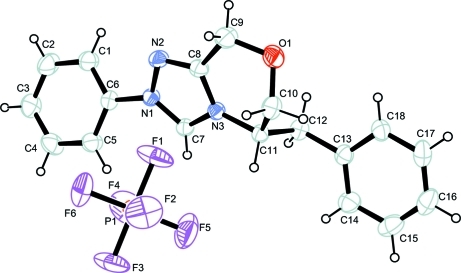
The molecular structure of (I), showing 30% probability displacement ellipsoids and the atomic numbering.

## supplementary crystallographic information

### Comment

Triazolium salts which can be used as the precursors of carbenes are widely used in asymmetric catalysis for the C—C bond formation reactions, such as benzoin reactions (Knight & Leeper 1998; Ma *et al.* 2008), Stetter reactions (Kerr *et al.* 2002; Readde Alaniz & Rovis 2005) and Diels-Alder reactions (Fisher *et al.* 2006) because of their excellent catalytic performance in umpolung aldehyde chemistry. Most of the research performed shows that chiral bicyclic 1,2,4-triazole carbenes have excellent enanselectivity because they have many bulkier groups and show weaker nucleophility than thiazolium, imidazolium and imidazolinium salts. The crystal structure of the title compound shows that N1—C7—N3 is a typical 3-center-4-electron bond (well known as the Pimentel-Rundle three-center model), because both N1—C7 (1.312 (3) Å) and N3—C7 (1.325 (2) Å) bond lengths are longer than the N2=C8 (1.292 (3) Å) double bond but shorter than other N—C single bonds (1.365 (3)–1.486 (3) Å). The dihedral angle between the phenyl (C1—C6) and triazolium ring (N1/N2/C8/N3/C7) is 27.8 °. In the crystal packing, weak P—F···π (*Cg* 1) interactions interconnect adjacent molecules at the same time that it provides to the stability of the crystal structure.

### Experimental

The title compound was prepared according to literature methods (Knight & Leeper 1998). A solution of 3-benzyl-5-ethoxy-3,6-dihydro-2*H*-1,4-oxazine (prepared from (*s*)-2-amino-3-phenylpropan-1-ol) as a colorless liquid was added dropwise to phenylhydrazine hydrochloride (1.44 g, 10 mmol) in methanol (3 ml). The mixture was then stirred for 30 min, followed by addition of triethyl orthoformate (7.4 g, 50 mmol). After being heated at 353 K for 10 h, the reaction mixture was cooled to room temperature and concentrated *in vacuo*. The resulting residue was purified by column chromatography on silica gel with elution with methanol and followed with anion exchange with ammonium hexafluorophosphate to afford the pure triazolium salt (I) as a white solid in 63% overall yield. Colourless crystals suitable for X-ray structural analysis were grown by slow evaporation of actone solution. ^1^H NMR (400 MHz, DMSO): δ 3.17 (1 H, dd, *J* = 10.4 Hz, 4.8 Hz, 1H), 3.19 (dd, *J* = 10.0 Hz, 4.8 Hz, 1H), 3.95–4.01 (m, 2H), 4.87–4.93 (m, 1H), 5.22 (2 d, *J* = 16 Hz, 16 Hz, 2H), 7.47–7.68 (m, 8H), 7.69–7.91 (m, 2H), 11.20 (s, 1H).

### Refinement

All H atoms were positioned geometrically and refined in the riding model approximation with C—H = 0.93, 0.97 or 0.98 Å, *U*_iso_(H) = 1.2*U*_eq_(C or N)

### Crystal data

**Table:**

C_18_H_18_N_3_O^+^·F_6_P^−^	*F*(000) = 448
*M**_r_* = 437.32	*D*_x_ = 1.506 Mg m^−^^3^
Monoclinic, *P*2_1_	Mo *K*α radiation, λ = 0.71073 Å
Hall symbol: P 2yb	Cell parameters from 3172 reflections
*a* = 11.4054 (13) Å	θ = 3.1–26.0°
*b* = 8.1243 (9) Å	µ = 0.21 mm^−^^1^
*c* = 11.8593 (14) Å	*T* = 297 K
β = 118.678 (2)°	Parallelepiped, colourless
*V* = 964.09 (19) Å^3^	0.53 × 0.42 × 0.32 mm
*Z* = 2	

### Data collection

**Table:**

Bruker SMART CCD area-detector diffractometer	3406 independent reflections
Radiation source: fine-focus sealed tube	2984 reflections with *I* > 2σ(*I*)
graphite	*R*_int_ = 0.022
φ and ω scans	θ_max_ = 26.0°, θ_min_ = 2.0°
Absorption correction: multi-scan (*SADABS*; Sheldrick, 1996)	*h* = −14→13
*T*_min_ = 0.89, *T*_max_ = 0.93	*k* = −9→10
5505 measured reflections	*l* = −12→14

### Refinement

**Table:**

Refinement on *F*^2^	Hydrogen site location: inferred from neighbouring sites
Least-squares matrix: full	H-atom parameters constrained
*R*[*F*^2^ > 2σ(*F*^2^)] = 0.039	*w* = 1/[σ^2^(*F*_o_^2^) + (0.075*P*)^2^] where *P* = (*F*_o_^2^ + 2*F*_c_^2^)/3
*wR*(*F*^2^) = 0.114	(Δ/σ)_max_ = 0.009
*S* = 1.11	Δρ_max_ = 0.20 e Å^−^^3^
3406 reflections	Δρ_min_ = −0.20 e Å^−^^3^
318 parameters	Extinction correction: *SHELXL97* (Sheldrick, 2008), Fc^*^=kFc[1+0.001xFc^2^λ^3^/sin(2θ)]^-1/4^
31 restraints	Extinction coefficient: 0.019 (3)
Primary atom site location: structure-invariant direct methods	Absolute structure: Flack (1983), 1368 Friedel pairs
Secondary atom site location: difference Fourier map	Flack parameter: 0.01 (10)

### Special details

**Table:**

Experimental. Even if the Flack parameter coming out of refinement appears quite trustable, the fact that the heaviest atomic species in the structure is P could suggest that the absolute structure determination could be thought as dubious. However, because the stereogenic carbon does not directly participate in the cyclocondensation, there is little risk for the racemization of the stereogenic carbon in this reaction. (Knight, *R. L*. & Leeper, F. J. (1998) *J. Chem. Soc., Perkin Trans. 1*, 1891–1893.) From starting material (*S*)-2-amino-3-phenylpropan-1-ol, it give (*S*)-5-benzyl-2-phenyl-6,8-dihydro-5*H*- [1,2,4]triazolo[3,4-*c*][1,4]oxazin-2-ium hexafluorophosphate as product, whose absolute configuration (*s*) is consistent with the absolute structure characterized by X-ray structure analysis.
Geometry. All e.s.d.'s (except the e.s.d. in the dihedral angle between two l.s. planes) are estimated using the full covariance matrix. The cell e.s.d.'s are taken into account individually in the estimation of e.s.d.'s in distances, angles and torsion angles; correlations between e.s.d.'s in cell parameters are only used when they are defined by crystal symmetry. An approximate (isotropic) treatment of cell e.s.d.'s is used for estimating e.s.d.'s involving l.s. planes.
Refinement. Refinement of *F*^2^ against ALL reflections. The weighted *R*-factor *wR* and goodness of fit *S* are based on *F*^2^, conventional *R*-factors *R* are based on *F*, with *F* set to zero for negative *F*^2^. The threshold expression of *F*^2^ > σ(*F*^2^) is used only for calculating *R*-factors(gt) *etc*. and is not relevant to the choice of reflections for refinement. *R*-factors based on *F*^2^ are statistically about twice as large as those based on *F*, and *R*- factors based on ALL data will be even larger.

### Fractional atomic coordinates and isotropic or equivalent isotropic displacement parameters (Å^2^)

**Table:**

	*x*	*y*	*z*	*U*_iso_*/*U*_eq_	Occ. (<1)
P1	0.79225 (6)	0.03833 (8)	0.45453 (6)	0.0488 (2)	
O1	0.67186 (18)	0.5581 (3)	0.70365 (16)	0.0669 (5)	
N1	0.69585 (16)	0.5547 (3)	0.34299 (15)	0.0428 (4)	
N2	0.59091 (18)	0.5715 (3)	0.36766 (18)	0.0514 (5)	
N3	0.77918 (16)	0.5257 (3)	0.54549 (15)	0.0422 (4)	
C1	0.5666 (3)	0.6481 (4)	0.1230 (3)	0.0637 (7)	
H1A	0.5131	0.7093	0.1469	0.076*	
C2	0.5404 (3)	0.6440 (5)	−0.0031 (3)	0.0788 (9)	
H2B	0.4675	0.7013	−0.0654	0.095*	
C3	0.6212 (3)	0.5557 (5)	−0.0376 (3)	0.0707 (8)	
H3A	0.6023	0.5529	−0.1231	0.085*	
C4	0.7283 (3)	0.4729 (5)	0.0528 (3)	0.0734 (9)	
H4A	0.7841	0.4162	0.0293	0.088*	
C5	0.7556 (3)	0.4719 (4)	0.1800 (3)	0.0636 (7)	
H5A	0.8279	0.4132	0.2418	0.076*	
C6	0.6733 (2)	0.5598 (3)	0.2126 (2)	0.0464 (5)	
C7	0.8076 (2)	0.5279 (4)	0.44951 (19)	0.0435 (5)	
H7A	0.8917	0.5132	0.4564	0.052*	
C8	0.6451 (2)	0.5535 (4)	0.4908 (2)	0.0486 (5)	
C9	0.5763 (3)	0.5573 (6)	0.5721 (2)	0.0713 (9)	
H9A	0.5209	0.6550	0.5519	0.086*	
H9B	0.5190	0.4616	0.5533	0.086*	
C10	0.7696 (3)	0.4335 (4)	0.7317 (2)	0.0579 (7)	
H10A	0.7261	0.3313	0.6908	0.069*	
H10B	0.8187	0.4152	0.8237	0.069*	
C11	0.8654 (2)	0.4849 (3)	0.6834 (2)	0.0447 (5)	
H11A	0.9229	0.3914	0.6904	0.054*	
C12	0.9534 (3)	0.6333 (3)	0.7548 (2)	0.0531 (6)	
H12A	1.0050	0.6667	0.7133	0.064*	
H12B	0.8971	0.7250	0.7510	0.064*	
C13	1.0474 (2)	0.5901 (3)	0.8942 (2)	0.0514 (6)	
C14	1.1674 (3)	0.5140 (4)	0.9300 (3)	0.0677 (8)	
H14A	1.1937	0.4904	0.8686	0.081*	
C15	1.2500 (3)	0.4720 (5)	1.0580 (4)	0.0796 (9)	
H15A	1.3321	0.4225	1.0819	0.095*	
C16	1.2119 (3)	0.5024 (4)	1.1490 (3)	0.0794 (10)	
H16A	1.2674	0.4726	1.2341	0.095*	
C17	1.0925 (3)	0.5766 (4)	1.1146 (3)	0.0710 (8)	
H17A	1.0662	0.5973	1.1764	0.085*	
C18	1.0103 (3)	0.6211 (4)	0.9885 (3)	0.0590 (7)	
H18A	0.9292	0.6726	0.9660	0.071*	
F1	0.7113 (5)	0.1769 (5)	0.4784 (7)	0.105 (2)	0.678 (8)
F2	0.7470 (6)	−0.0824 (7)	0.5298 (6)	0.115 (2)	0.678 (8)
F3	0.8763 (5)	−0.0945 (6)	0.4304 (8)	0.118 (3)	0.678 (8)
F4	0.8458 (8)	0.1611 (7)	0.3921 (7)	0.126 (3)	0.678 (8)
F5	0.9187 (4)	0.0816 (12)	0.5876 (4)	0.132 (3)	0.678 (8)
F6	0.6692 (5)	−0.0133 (10)	0.3319 (4)	0.128 (2)	0.678 (8)
F1'	0.767 (2)	0.125 (3)	0.5475 (17)	0.214 (11)	0.322 (8)
F2'	0.6898 (13)	−0.0908 (11)	0.433 (2)	0.142 (7)	0.322 (8)
F3'	0.811 (3)	−0.036 (4)	0.350 (2)	0.280 (14)	0.322 (8)
F4'	0.8949 (12)	0.1741 (13)	0.476 (2)	0.154 (9)	0.322 (8)
F5'	0.9061 (10)	−0.069 (2)	0.542 (2)	0.174 (9)	0.322 (8)
F6'	0.6823 (13)	0.138 (2)	0.3481 (18)	0.192 (8)	0.322 (8)

### Atomic displacement parameters (Å^2^)

**Table:**

	*U*^11^	*U*^22^	*U*^33^	*U*^12^	*U*^13^	*U*^23^
P1	0.0462 (3)	0.0449 (3)	0.0583 (4)	−0.0032 (3)	0.0276 (3)	−0.0032 (3)
O1	0.0668 (10)	0.0884 (15)	0.0578 (10)	0.0000 (12)	0.0398 (9)	−0.0050 (11)
N1	0.0398 (8)	0.0493 (11)	0.0410 (9)	−0.0007 (10)	0.0207 (7)	0.0010 (9)
N2	0.0407 (9)	0.0657 (16)	0.0500 (11)	0.0068 (10)	0.0234 (8)	0.0035 (10)
N3	0.0418 (8)	0.0444 (11)	0.0410 (9)	0.0000 (10)	0.0203 (7)	0.0004 (9)
C1	0.0640 (16)	0.0757 (19)	0.0531 (15)	0.0131 (15)	0.0296 (14)	0.0078 (13)
C2	0.0738 (19)	0.104 (3)	0.0513 (16)	0.015 (2)	0.0243 (15)	0.0201 (17)
C3	0.0831 (18)	0.085 (2)	0.0498 (14)	−0.002 (2)	0.0368 (14)	0.0091 (16)
C4	0.092 (2)	0.083 (2)	0.0690 (18)	0.0091 (18)	0.0574 (18)	0.0011 (16)
C5	0.0673 (16)	0.0746 (18)	0.0560 (15)	0.0157 (15)	0.0353 (13)	0.0103 (13)
C6	0.0459 (10)	0.0514 (15)	0.0429 (11)	−0.0081 (12)	0.0220 (9)	0.0001 (11)
C7	0.0395 (10)	0.0474 (13)	0.0451 (11)	−0.0023 (11)	0.0216 (9)	−0.0006 (10)
C8	0.0448 (11)	0.0535 (14)	0.0520 (12)	0.0022 (12)	0.0269 (10)	−0.0003 (12)
C9	0.0583 (13)	0.110 (3)	0.0584 (15)	0.012 (2)	0.0380 (13)	0.0084 (19)
C10	0.0607 (15)	0.0610 (17)	0.0487 (14)	−0.0096 (13)	0.0237 (12)	0.0041 (12)
C11	0.0463 (12)	0.0444 (13)	0.0402 (11)	0.0020 (9)	0.0182 (10)	0.0008 (9)
C12	0.0546 (14)	0.0538 (15)	0.0494 (13)	−0.0061 (12)	0.0237 (12)	0.0018 (11)
C13	0.0511 (13)	0.0465 (14)	0.0512 (13)	−0.0076 (10)	0.0201 (11)	−0.0039 (10)
C14	0.0515 (13)	0.071 (2)	0.0762 (18)	−0.0059 (14)	0.0270 (13)	−0.0082 (15)
C15	0.0495 (15)	0.072 (2)	0.091 (2)	−0.0005 (15)	0.0126 (15)	0.0044 (17)
C16	0.079 (2)	0.064 (2)	0.0590 (17)	−0.0103 (16)	0.0041 (16)	0.0011 (14)
C17	0.092 (2)	0.064 (2)	0.0467 (14)	−0.0145 (16)	0.0250 (14)	−0.0090 (13)
C18	0.0632 (15)	0.0567 (16)	0.0523 (14)	−0.0026 (13)	0.0240 (13)	−0.0087 (12)
F1	0.084 (3)	0.054 (2)	0.219 (7)	−0.0148 (17)	0.107 (4)	−0.037 (3)
F2	0.125 (4)	0.104 (4)	0.137 (4)	−0.005 (3)	0.081 (3)	0.047 (3)
F3	0.079 (3)	0.072 (3)	0.216 (7)	0.009 (2)	0.083 (4)	−0.037 (4)
F4	0.190 (7)	0.091 (4)	0.167 (5)	−0.017 (4)	0.143 (5)	0.023 (4)
F5	0.076 (2)	0.214 (8)	0.081 (2)	−0.029 (4)	0.0164 (18)	−0.037 (3)
F6	0.098 (3)	0.163 (6)	0.073 (2)	−0.015 (3)	0.002 (2)	−0.033 (3)
F1'	0.30 (2)	0.26 (2)	0.156 (13)	−0.078 (17)	0.172 (16)	−0.120 (13)
F2'	0.100 (8)	0.052 (5)	0.33 (2)	−0.035 (5)	0.145 (12)	−0.062 (10)
F3'	0.34 (3)	0.40 (4)	0.237 (19)	−0.08 (2)	0.24 (2)	−0.16 (2)
F4'	0.072 (6)	0.075 (6)	0.34 (3)	−0.024 (5)	0.120 (12)	−0.059 (12)
F5'	0.085 (6)	0.148 (13)	0.218 (16)	0.027 (8)	0.015 (9)	0.130 (13)
F6'	0.119 (9)	0.163 (14)	0.181 (14)	0.025 (10)	−0.017 (9)	0.086 (12)

### Geometric parameters (Å, °)

**Table:**

P1—F1'	1.446 (10)	C4—C5	1.386 (4)
P1—F2'	1.498 (7)	C4—H4A	0.9300
P1—F5'	1.495 (7)	C5—C6	1.375 (4)
P1—F3'	1.480 (10)	C5—H5A	0.9300
P1—F6'	1.516 (8)	C7—H7A	0.9300
P1—F6	1.517 (4)	C8—C9	1.508 (3)
P1—F4	1.533 (4)	C9—H9A	0.9700
P1—F4'	1.539 (9)	C9—H9B	0.9700
P1—F1	1.565 (3)	C10—C11	1.515 (3)
P1—F3	1.559 (4)	C10—H10A	0.9700
P1—F2	1.570 (4)	C10—H10B	0.9700
P1—F5	1.584 (4)	C11—C12	1.537 (3)
O1—C9	1.410 (3)	C11—H11A	0.9800
O1—C10	1.422 (4)	C12—C13	1.518 (3)
N1—C7	1.312 (3)	C12—H12A	0.9700
N1—N2	1.369 (2)	C12—H12B	0.9700
N1—C6	1.442 (3)	C13—C14	1.371 (4)
N2—C8	1.292 (3)	C13—C18	1.395 (4)
N3—C7	1.325 (2)	C14—C15	1.391 (4)
N3—C8	1.364 (3)	C14—H14A	0.9300
N3—C11	1.486 (3)	C15—C16	1.365 (5)
C1—C6	1.373 (4)	C15—H15A	0.9300
C1—C2	1.377 (4)	C16—C17	1.361 (5)
C1—H1A	0.9300	C16—H16A	0.9300
C2—C3	1.376 (5)	C17—C18	1.379 (4)
C2—H2B	0.9300	C17—H17A	0.9300
C3—C4	1.356 (5)	C18—H18A	0.9300
C3—H3A	0.9300		
			
F1'—P1—F2'	92.7 (10)	C4—C5—H5A	120.8
F1'—P1—F5'	100.2 (14)	C1—C6—C5	121.7 (2)
F2'—P1—F5'	93.2 (9)	C1—C6—N1	118.7 (2)
F1'—P1—F3'	174.1 (17)	C5—C6—N1	119.5 (2)
F2'—P1—F3'	89.3 (11)	N1—C7—N3	107.70 (17)
F5'—P1—F3'	85.2 (12)	N1—C7—H7A	126.2
F1'—P1—F6'	89.4 (11)	N3—C7—H7A	126.2
F2'—P1—F6'	88.0 (9)	N2—C8—N3	112.03 (18)
F5'—P1—F6'	170.3 (13)	N2—C8—C9	127.4 (2)
F3'—P1—F6'	85.1 (15)	N3—C8—C9	120.6 (2)
F1'—P1—F4'	86.6 (10)	O1—C9—C8	110.13 (19)
F2'—P1—F4'	178.6 (6)	O1—C9—H9A	109.6
F5'—P1—F4'	88.1 (7)	C8—C9—H9A	109.6
F3'—P1—F4'	91.3 (11)	O1—C9—H9B	109.6
F6'—P1—F4'	90.9 (9)	C8—C9—H9B	109.6
F6—P1—F1	91.0 (3)	H9A—C9—H9B	108.1
F4—P1—F1	91.5 (3)	O1—C10—C11	110.0 (2)
F6—P1—F1	91.0 (3)	O1—C10—H10A	109.7
F4—P1—F1	91.5 (3)	C11—C10—H10A	109.7
F6—P1—F3	90.0 (3)	O1—C10—H10B	109.7
F4—P1—F3	86.5 (4)	C11—C10—H10B	109.7
F1—P1—F3	177.8 (3)	H10A—C10—H10B	108.2
F6—P1—F2	88.1 (3)	N3—C11—C10	105.10 (19)
F4—P1—F2	175.1 (4)	N3—C11—C12	110.14 (19)
F1—P1—F2	87.9 (3)	C10—C11—C12	114.0 (2)
F3—P1—F2	94.1 (4)	N3—C11—H11A	109.2
F6—P1—F5	175.9 (5)	C10—C11—H11A	109.2
F4—P1—F5	87.3 (3)	C12—C11—H11A	109.2
F1—P1—F5	89.7 (3)	C13—C12—C11	110.6 (2)
F3—P1—F5	89.5 (3)	C13—C12—H12A	109.5
F2—P1—F5	87.8 (4)	C11—C12—H12A	109.5
C9—O1—C10	111.0 (2)	C13—C12—H12B	109.5
C7—N1—N2	110.85 (16)	C11—C12—H12B	109.5
C7—N1—C6	128.92 (17)	H12A—C12—H12B	108.1
N2—N1—C6	120.16 (17)	C14—C13—C18	118.4 (3)
C8—N2—N1	103.74 (17)	C14—C13—C12	121.3 (2)
C7—N3—C8	105.68 (17)	C18—C13—C12	120.3 (2)
C7—N3—C11	130.00 (18)	C13—C14—C15	120.0 (3)
C8—N3—C11	124.03 (17)	C13—C14—H14A	120.0
C6—C1—C2	118.5 (3)	C15—C14—H14A	120.0
C6—C1—H1A	120.7	C16—C15—C14	120.9 (3)
C2—C1—H1A	120.7	C16—C15—H15A	119.6
C3—C2—C1	120.6 (3)	C14—C15—H15A	119.6
C3—C2—H2B	119.7	C17—C16—C15	119.7 (3)
C1—C2—H2B	119.7	C17—C16—H16A	120.1
C4—C3—C2	120.1 (2)	C15—C16—H16A	120.1
C4—C3—H3A	120.0	C16—C17—C18	120.2 (3)
C2—C3—H3A	120.0	C16—C17—H17A	119.9
C3—C4—C5	120.7 (3)	C18—C17—H17A	119.9
C3—C4—H4A	119.7	C17—C18—C13	120.8 (3)
C5—C4—H4A	119.7	C17—C18—H18A	119.6
C6—C5—C4	118.4 (3)	C13—C18—H18A	119.6
C6—C5—H5A	120.8		

### Hydrogen-bond geometry (Å, °)

**Table:**

*D*—H···*A*	*D*—H	H···*A*	*D*···*A*	*D*—H···*A*
P1—F1···Cg1^i^	1.57 (1)	3.03	4.235 (11)	132
P1—F2···Cg1	1.57 (1)	3.19	4.102 (11)	115
P1—F2'···Cg1	1.50 (1)	2.93	4.102 (11)	133

Symmetry codes: (i) *x*, *y*+1, *z*.
